# Electronic health record (EHR)-based PROMIS measures among neurology clinic decedents and survivors: a retrospective cohort analysis

**DOI:** 10.1186/s12955-023-02176-0

**Published:** 2023-08-21

**Authors:** Natalie C. Ernecoff, Rebecca Weir, Anthony Rodriguez, Lucy B. Schulson, Maria Orlando Edelen, Janel Hanmer

**Affiliations:** 1 RAND Corporation, Santa Monica, USA; 2 Bringham and Women’s Hospital, Boston, USA; 3 Division of General Internal Medicine, School of Medicine, University of Pittsburgh, Pittsburgh, USA

## Abstract

**Background:**

In addition to their standard use to assess real-time symptom burden, patient-reported outcomes (PROs), such as the Patient-Reported Outcomes Measurement Information System (PROMIS), measures offer a potential opportunity to understand when patients are experiencing meaningful clinical decline. If PROs can be used to assess decline, such information can be used for informing medical decision making and determining patient-centered treatment pathways. We sought to use clinically implemented PROMIS measures to retrospectively characterize the final PROMIS report among all patients who completed at least one PROMIS assessment from December 2017-March 2020 in one large health system, stratified by decedents vs. survivors. We conducted a retrospective cohort analysis of decedents (N = 1,499) who received care from outpatient neurology clinical practice within a single, large health system as part of usual care. We also compared decedents to survivors (360 + days before death; N = 49,602) on PROMIS domains and PROMIS-Preference (PROPr) score, along with demographics and clinical characteristics. We used electronic health record (EHR) data with built-in PROMIS measures. Linear regressions assessed differences in PROMIS domains and aggregate PROPr score by days before death of the final PROMIS completion for each patient.

**Results:**

Among decedents in our sample, in multivariable regression, only fatigue (range 54.48–59.38, p < 0.0029) and physical function (range 33.22–38.38, p < 0.0001) demonstrated clinically meaningful differences across time before death. The overall PROPr score also demonstrated statistically significant difference comparing survivors (0.19) to PROPr scores obtained 0–29 days before death (0.29, p < 0.0001).

**Conclusions:**

Although clinic completion of PROMIS measures was near universal, very few patients had more than one instance of PROMIS measures reported, limiting longitudinal analyses. Therefore, patient-reported outcomes in clinical practice may not yet be robust enough for incorporation in prediction models and assessment of trajectories of decline, as evidenced in these specialty clinics in one health system. PROMIS measures can be used to effectively identify symptoms and needs in real time, and robust incorporation into EHRs can improve patient-level outcomes, but further work is needed for them to offer meaningful inputs for defining patient trajectories near the end of life.

**Plain English Summary:**

Assessing symptom burden provides an opportunity to understand clinical decline, particularly as people approach the end of life. We sought to understand whether symptoms reported by patients can be used to assess decline in health. Such information can inform decision-making about care and treatments. Of eight symptoms that we assessed, patient reports of fatigue and physical function were associated with clinical decline, as was an overall score of symptom burden. Because few symptoms were associated with decline, patient-reported outcomes in clinical practice may not yet be robust enough for incorporation in prediction models and assessment of trajectories of decline.

## Background

Identifying clinical decline before death is critical because decline and associated prognosis are essential elements of patient-centered medical decision-making and delivery of care consistent with patient goals and values (i.e., goal-concordant care) [1]. For example, as many patients approach the end of life, they and their families (especially for those who are cognitively incapacitated) shift goals to improving comfort and discontinuing treatments, as those treatments lose potential effectiveness in the context of progressing disease. One goal-driven treatment option is hospice, which requires a prognosis of ≤ 6 months, and many patients and families decline other intensive treatments, such as cardiopulmonary resuscitation (CPR) or ventilator support [2–4]. Therefore, it is important to explore opportunities to identify patient decline preceding death more precisely.

The Patient-Reported Outcomes Measurement Information System (PROMIS) offers an opportunity to identify patient-reported outcomes (PROs) that may be associated with clinical decline among people near the end of life [5]. Diehr et al. characterized longitudinal history of disease including acute events and tracked co-occurring patient-reported health; they found that declines in patient-perceived health precipitate acute events, and an additional drop in score resulted from the acute event [6, 7]. Evidence indicates that terminal decline begins 3–5 years before death, thought additional research is needed to identifying trajectories meaningful to health care delivery and practice [8]. One approach, integrating real-world outpatient assessment of symptom trajectories before death via PROMIS, has yet to be evaluated.

Therefore, we conducted a pragmatic study of real-world clinical implementation of PROMIS measures to retrospectively characterize differences in reported health-related quality of life before death among people who received care in neurology clinics across one large health system.

## Methods

### Study design

We used retrospective, structured electronic health record (EHR) data at an academic medical center in Pennsylvania to characterize clinical differences in PROMIS scores between decedents and survivors, and across decedents who completed PROMIS measures in the last year of life, to determine any potential patterns in PROMIS before death. Because PROMIS was systematically implemented across outpatient neurology within the health system, nearly all observations were obtained in standard neurology clinics, which had 82% completion rates during the study period.

Study procedures were approved by the RAND Corporation and University of Pittsburgh Institutional Review Boards.

### Setting & data source

We extracted EHR data for all patients who completed at least one PROMIS assessment from December 2017-March 2020. We stratified by survivors (N = 49,602) and decedents (N = 1,499) during the study period. We included all people with a cognitive function score from the PROMIS-16 in neurology clinics. We then subset to patients who had at least one PROMIS assessment, at least one PROMIS cognitive assessment, demographic data, diagnosis data ≤ 180 days before the date of the first PROMIS assessment, and who were at least 18 years old at the time of the first PROMIS assessment.

The EHR automatically assigns the PROMIS-16 at all neurology visit types. It is sent to the patient portal 7 days before the visit. If the patient or their proxy does not complete the questionnaire on the portal, the front desk staff provide a tablet computer in the waiting room for completion. If patients are unable or unwilling to use the tablet, questions are asked verbally by staff during rooming. All responses are immediately scored and available in the EHR.

### Variables

Demographic & Clinical Characteristics: We obtained data on patient demographics (age, gender, race) and clinical characteristics (International Classification of Diseases (ICD)-10 diagnoses, PROMIS cognitive function) [9]. For each patient, we identified all ICD-10 diagnosis codes from clinical encounters that occurred within the six months prior to the first PROMIS assessment. We measured comorbidity burden using diagnoses from the Charlson Comorbidity Index with available EHR data, which included chronic pulmonary disease, diabetes, cancer, peripheral vascular disease, renal disease, hemiplegia/paraplegia, congestive heart failure, liver disease, myocardial infarction, and rheumatic disease [10]. In assessing comorbidities, we also included depression and hypertension diagnoses from the Elixhauser, due to their clinical association with cognitive impairment (CI) [11]. We used the Clinical Classifications Software Refined (CCS-R) and consultation with a physician (LBS) to categorize patients as having no CI, possible CI, or definite CI based on ICD-10 diagnoses.

Mortality: We obtained mortality indicators from vital statistics linked to EHR data. Among decedents, we defined time before death of the last PROMIS assessment categorically at 30-day intervals up to 90 days, then 90-day intervals up to 359 days. We aggregated “survivors” into one category those who died ≥ 360 days after most recent PROMIS assessment.

Symptom and Functional Assessment: We assessed symptoms and function with 16 questions from eight PROMIS domains, with 2 items per domain. The 16 items yielded scores for each of the eight domains: anxiety, cognitive function, depression, fatigue, pain interference, physical function, sleep disturbance, and ability to participate in social roles and activities. The PROMIS-Preference (PROPr) Score is the aggregate of seven of the domains (excluding anxiety) and has a meaningful clinically important difference of 0.04 [12, 13].

### Analysis

We summarized demographic, clinical, and PROMIS descriptive characteristics for survivors and decedents with univariate and bivariate statistics. Based on inclusion criteria, we conducted a complete-case analysis. We ran chi-square tests and t-tests to compare decedents and survivors on demographic and clinical characteristics. We ran linear regressions adjusting for age, gender, race, and partial Charlson Comorbidity Score to compare decedents and survivors on PROMIS domains and PROPr score. Among decedents, we assessed PROMIS domain and PROPr scores by days before death of the final PROMIS completion using linear regression adjusting for the same covariates. We note that the analyses on the full population of decedents and survivors had a denominator of 51,101, while the analyses on the decedents had a denominator of 1,499. For t-tests using the full sample, we had 80% power to detect a Cohen’s d of 0.07345, accounting for the allocation ratio (N2/N1) resulting in uneven group sizes. For test among decedents, we have 80% power to detect an effect size of 0.0086. Thus, we are extremely powered to detect all effects, and our interpretations account for meaningful clinically important difference. All analyses were conducted in SAS 9.4.

## Results

### Participant characteristics

We identified 51,101 patients with at least one PROMIS assessment, including 1,499 decedents. Compared to survivors, decedents were less likely to be female (45.36% vs. 60.74%, p < 0.0001), and were older (mean 70.44 vs. 53.63, p < 0.0001); there was no difference by race. As expected, decedents were more likely thank survivors to have all comorbidities, including cognitive impairment (all p < 0.0001). Complete characteristics for survivors and decedents with corresponding chi-square and t-tests are presented in Table 1.

**Table 1:** Patient characteristics, summarized by total and decedents vs. survivors, and chi-square tests and t-tests comparing decedents to survivors

Characteristics n(%)	Total N = 51,101	Decedents N = 1,499	Survivors N = 49,602	P-value
Gender				< 0.0001
Female	30,807 (60.29)	680 (45.36)	30,127 (60.74)	
Male	20,294 (39.71)	819 (54.64)	19,475 (39.26)	
Race				
White	44,638 (89.08)	1,322 (89.20)	43,316 (89.08)	0.1276
Black	4,607 (9.19)	144 (9.72)	4,463 (9.18)	
Other	863 (1.72)	16 (1.08)	847 (1.74)	
Age (Mean, SD)	54.12 (17.77)	70.44 (13.50)	53.63 (17.65)	< 0.0001
Cognitive Impairment (CI)				
None	27,886 (54.57)	566 (37.76)	27,320 (55.08)	< 0.0001
Possible	17,773 (34.78)	511 (34.09)	17,262 (34.80)	
Definite	5,442 (10.65)	422 (28.15)	5,020 (10.12)	
Comorbidities				
Chronic pulmonary disease	6,408 (12.54)	353 (23.55)	6,055 (12.21)	< 0.0001
Diabetes (w/out complication)	5,300 (10.37)	335 (22.35)	4,965 (10.01)	< 0.0001
Cancer	3,385 (6.62)	426 (28.42)	2,959 (5.97)	< 0.0001
Diabetes (w/complication)	2,905 (5.68)	249 (16.61)	2,656 (5.35)	< 0.0001
Dementia	2,716 (5.31)	338 (22.55)	2,378 (4.79)	< 0.0001
Peripheral vascular disease	2,429 (4.75)	237 (15.81)	2,192 (4.42)	< 0.0001
Renal disease	2,288 (4.48)	273 (18.21)	2,015 (4.06)	< 0.0001
Hemiplegia/paraplegia	2,036 (3.98)	130 (8.67)	1,906 (3.84)	< 0.0001
Congestive heart failure	1,987 (3.89)	258 (17.21)	1,729 (3.49)	< 0.0001
Mild liver disease	1,549 (3.03)	106 (7.07)	1,443 (2.91)	< 0.0001
Myocardial infarction	1,425 (2.79)	132 (8.81)	1,293 (2.61)	< 0.0001
Rheumatic disease	1,407 (2.75)	70 (4.67)	1,337 (2.70)	< 0.0001
Metastatic carcinoma	577 (1.13)	151 (10.07)	426 (0.86)	< 0.0001
Moderate/severe liver disease	179 (0.35)	28 (1.87)	151 (0.30)	< 0.0001
Depression	8,107 (15.86)	332 (22.15)	7,775 (15.67)	< 0.0001
Hypertension	15,448 (30.23)	850 (56.70)	14,598 (29.43)	< 0.0001

### PROMIS completion

Most patients (58.82% of survivors and 68.71% of decedents) completed PROMIS measures at only one timepoint during the study period. The last PROMIS assessment before death for each decedent was most often in the 3–9 months preceding death; there was a drop-off in PROMIS completion in the last two months before death.

### PROMIS scores preceding death

All PROMIS domain scores (anxiety, cognitive function, depression, fatigue, physical function, and ability to participate in social roles and activities) except pain interference and sleep disturbance were significantly worse for decedents compared to survivors in linear regressions controlling for demographic and clinical characteristics. Only physical function (36.33 in decedents vs. 44.23 in survivors, p < 0.0001) and ability to participate in social roles and activities (44.39 in decedents vs. 50.17 in survivors, p < 0.0001) demonstrated a minimal clinically important difference. The overall PROPr score was also statistically and clinically significantly lower among decedents (0.25) compared to survivors (0.36, p < 0.0001) (Table 2).

**Table 2:** Summary of most recent PROMIS and PROPr scores, all PROMIS domains, total and stratified by decedents vs. survivors, including linear regressions comparing decedents to survivors

PROMIS Domain Mean (SD)	Total N = 51,101	Decedents N = 1,499	Survivors N = 49,602	Effect Size (unadjusted mean difference)	p-value
Anxiety	54.58 (9.66)	56.01 (9.73)	54.54 (9.65)	0.15	< 0.0001*
Cognitive Function**	48.87 (8.66)	47.49 (8.99)	48.91 (8.65)	0.16	< 0.0001*
Depression	51.70 (9.18)	53.60 (9.41)	51.65 (9.16)	0.21	< 0.0001*
Fatigue	53.57 (10.44)	55.66 (10.12)	53.50 (10.44)	0.21	< 0.0001*
Pain Interference	56.37 (10.09)	56.75 (10.30)	56.36 (10.08)	0.04	0.0796
Physical Function**	44.00 (10.10)	36.33 (10.72)	44.23 (9.99)	0.79	< 0.0001*
Sleep Disturbance	52.66 (8.26)	52.00 (8.12)	52.68 (8.26)	0.08	0.5160
Ability to Participate in Social Roles and Activities**	50.00 (9.40)	44.39 (9.80)	50.17 (9.33)	0.62	< 0.0001*
PROPr Score**	0.35 (0.24)	0.25 (0.20)	0.36 (0.24)	0.46	< 0.0001*

Linear regressions controlled for age, gender, race, and partial Charlson Comorbidity Score

*p < 0.05

**For these domain scores, higher scores indicate better quality of life. Higher score indicates higher presence of domain. Higher PROPr score indicates better overall quality of life

Among all decedents, five of the eight PROMIS domains were statistically significant in linear regressions when comparing across those who had a final PROMIS assessment closer vs. further from the time of death (Table 3): cognitive function (p = 0.0031), depression (p = 0.0161), fatigue (p = 0.0029), physical function (p < 0.0001), and ability to participate in social roles and activities (p = 0.0004). However, only fatigue (range 54.48–59.38) and physical function (range 33.22–38.38) demonstrated clinically meaningful differences [13]. The overall PROPr score was also statistically significantly lower among decedents 0–29 days before death (0.19) compared to those 360 + days from death (0.29, p < 0.0001) in the multivariable model.

**Table 3:** Among decedents (N = 1,499) who died within the study period, linear regressions for PROMIS domains and PROPr score by days before death

PROMIS Domain	Maximum Mean	Category of days before death that was associated with maximum score	Minimum Mean	Category of days before death that was associated with minimum score	p-value
Anxiety	56.67	**270–359 days**	55.01	**360 + days**	0.2394
Cognitive Function**	48.97	**360 + days**	46.15	**30–59 days**	0.0031*
Depression	55.58	**30–59 days**	52.28	**360 + days**	0.0161*
Fatigue	59.38	**0–29 days**	54.48	**270–359 days**	0.0029*
Pain Interference	57.99	**0–29 days**	55.67	**60–89 days**	0.4767
Physical Function**	38.38	**360 + days**	33.22	**0–29 days**	< 0.0001*
Sleep Disturbance	53.83	**0–29 days**	50.60	**60–89 days**	0.0845
Ability to Participate in Social Roles and Activities**	46.09	**360 + days**	42.18	**60–89 days**	0.0004*
PROPr Score**	0.29	**360 + days**	0.19	**0–29 days**	< 0.0001*

Linear regressions controlled for age, gender, race, and partial Charlson Comorbidity Score

The categories for the days before death variable were 0–29 days, 30–59 days, 60–89 days, 90–179 days, 180–269 days, 270–359 days, and 360 + days

*p < 0.05

**For these domain scores, higher scores indicate better quality of life. Higher score indicates higher presence of domain. Higher PROPr score indicates better overall quality of life

## Discussion

While models can help guide clinician practice, predictive models are notably limited in their ability to guide decision making for individual patients due to inherent error and uncertainty at the patient level. In a pragmatic study of real-world clinical implementation of PROMIS measures, we found little practical association between PROMIS scores and mortality. We identified three reasons the PROMIS may not be associated with clinically meaningful differences in most measured domains. First, upon assessment of available longitudinal data, we found very few repeat measures, even in clinics in which nearly all patients completed PROMIS measures at each visit. This may be due to longer-than-expected time between appointments or frequent appointment cancelation, particularly as people approach the end of life. Second, in absolute terms, PROMIS reporting in clinic was even lower in the two months preceding death than in prior months. This may be driven by acute decline (including hospitalization) that keeps patients from attending outpatient neurology appointments and completing PROMIS measures. Likewise, increasing symptom burden may lead to canceled appointments, whereupon PROMIS reports might indicate significant symptom burden. Further, patients may enroll in hospice and appropriately discontinue their usual clinical follow-up before death. Third, though sensitive to longitudinal change in empirical settings, changes in PROMIS may not be sensitive enough to be associated with clinically meaningful decline [14]. It is important that clinical screeners do not over-promise what they are able to reasonably support: while PROMIS can guide symptom management, it may not be able to predict or anticipate outcomes with any reliability. Notable, the domains that were associated with clinically meaningful differences before death were fatigue and physical function, two overarching domains that have been demonstrated to decline in the end-stages of serious illness, along with other functional status [15–17]. PROs continue to be one way to assess functional status and fatigue as predictors of mortality.

These findings are important both for clinical prediction and pragmatic research that uses PROs as part of clinical evaluation. Of note, PROMIS was not designed for prediction; it was designed to screen symptoms and prompt a conversation between clinicians and patients about goals of care and effective approaches to symptom management at the patient level [18]. At the population level, the signal is not as pronounced as researchers might anticipate. Although the denominator could be redefined to improve precision and accuracy in the sample, that approach may lead to less pragmatic, generalizable application.

Future work can test the effects of incorporating PROMIS measures, particularly for domains of functional status and fatigue, with other prognostic domains to build more robust prediction models of disease trajectories. Even if a prediction works well in a sample, attention must be paid to whether the prediction is clinically meaningful for care and decision making at the patient level. Still, it remains important to be mindful of the degree to which PROMIS measures are (or are not) incorporated into traditional data sources (e.g., EHRs) used for prediction. Generalizability of models that include PROMIS is limited to institutions that incorporate such measures. To that end, efforts to incorporate patient-reported outcomes into EHRs continues to be of high importance [19].

### Limitations

This study has several limitations. First, it was conducted in a single academic medical system with limited patient diversity and may lack generalizability to other health systems with a differing patient mix. Although longitudinal data were limited for this analysis and prevented a quasi-experimental design, these EHR data reflect data available in real-world clinical practice, thereby indicating that patient-reported outcomes may indeed be best used for real-time symptom assessment and treatment rather than for incorporation into prediction models.

## Conclusions

Though screeners such as PROMIS provide clinical utility for identifying and treating symptoms, their predictive power was limited in a system of neurology clinics. PROMIS continues to provide real-time symptom screening to support clinicians in identifying and meeting patient needs to support quality of life and may provide an opportunity to capture patient-reported decline, particular with respect to fatigue and functional status, near the end of life.

## Data Availability

The datasets generated and/or analysed during the current study are not publicly available because they are protected health information (PHI) from the electronic health record (EHR).
